# Game Development as an Educational Strategy in Dentistry

**DOI:** 10.1002/jdd.70053

**Published:** 2025-09-25

**Authors:** Isabella Zacarin Guiati

**Affiliations:** ^1^ Department of Biomaterials and Oral Biology University of São Paulo São Paulo Brazil

## 1 Problem

In evening programs, most students work during the day and attend classes at night, which means they often arrive at the university already tired and, in many cases, lacking the energy to fully engage in learning. Engaging students in the evening Dentistry program has been a major challenge for teachers, as the combination of the complex content taught in the Head and Neck Anatomy course and the fatigue of students, who have worked all day, creates a scenario that hinders effective learning. To address this challenge, it's essential to adopt methods that deepen previously covered content without overloading students. Such approaches must be both enjoyable and engaging to be effective.

## 2 Solution

For an evening class in a private dental school located in the interior of São Paulo state, Brazil, gamification was proposed to enhance student engagement. Gamification, based on experiential learning theory, can be defined as the introduction of game elements into a nongame context to increase student engagement and motivation [1]. Educational games have proven to be effective learning tools for undergraduate students, improving knowledge and skills, as well as increasing engagement and motivation [2]. In the gamification activity proposed for this class, students were encouraged to create games based on topics covered during the course (2024). They were divided into groups of eight and were given specific guidelines for developing the games.

## 3 Results

**FIGURE 1:**
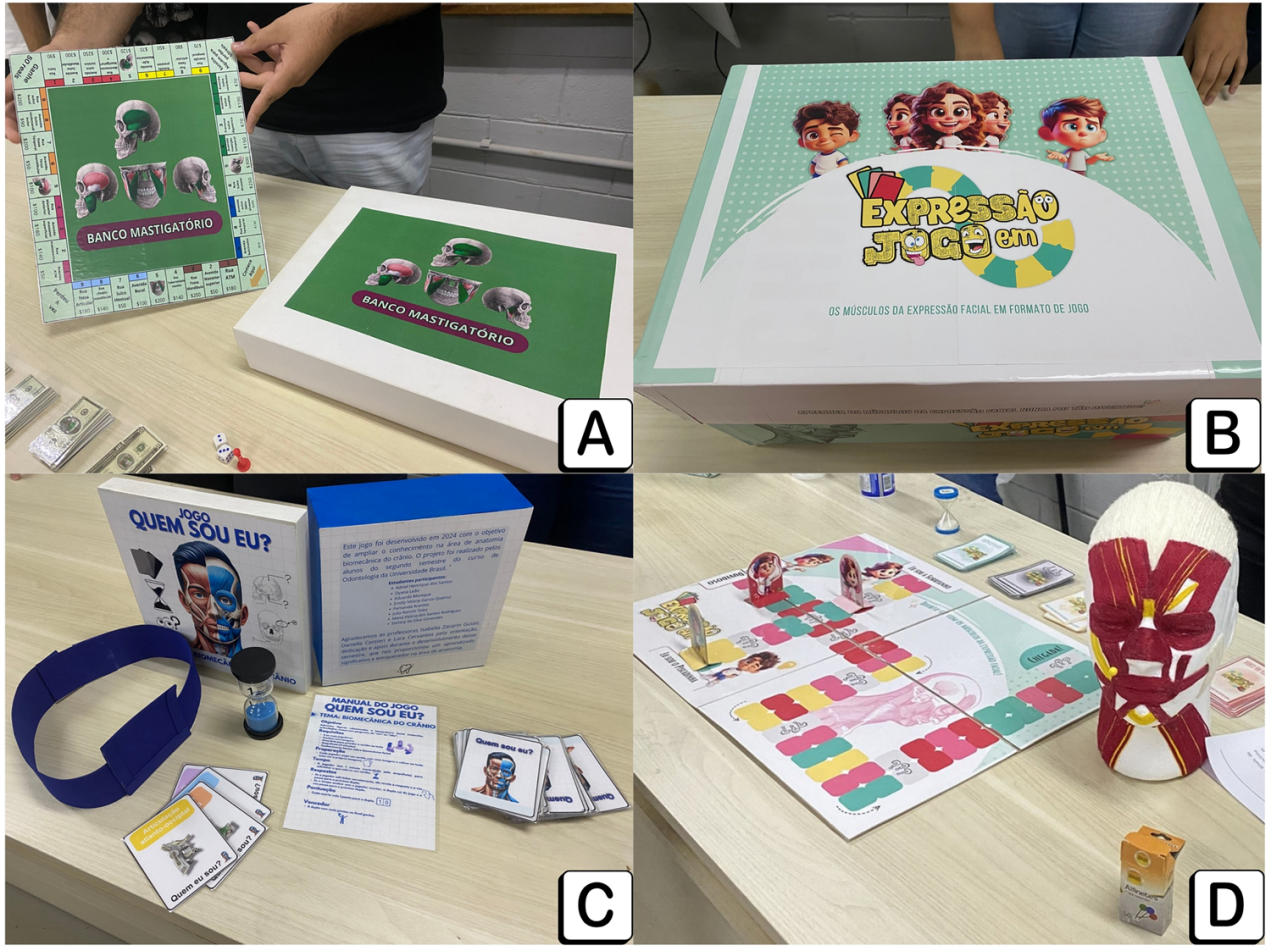
Games presented by students in the Head and Neck Anatomy course in 2024. (A) Game related to the topic “Muscles of Mastication,” (B) Game related to the topic “Facial Expression Muscles,” (C) Game related to the topic “Cranial Biomechanics,” (D) Game related to the topic "Salivary Glands.”

The games presented showed excellent graphic and academic quality and were described as “surprising” by faculty members (Figure 1). A questionnaire based on the Likert scale was administered to assess student satisfaction after the in‐class presentation of the games. All data presented here were authorized by the students. When asked whether the development of games during the Head and Neck Anatomy course facilitated their learning, 91.7% of students agreed. In response to the statement “The use of games in anatomy class reinforced content already covered during the semester,” 94.6% of students agreed. Regarding enjoyment, 81.1% of students reported that learning through game development was more enjoyable than through traditional anatomy.

**FIGURE 2:**
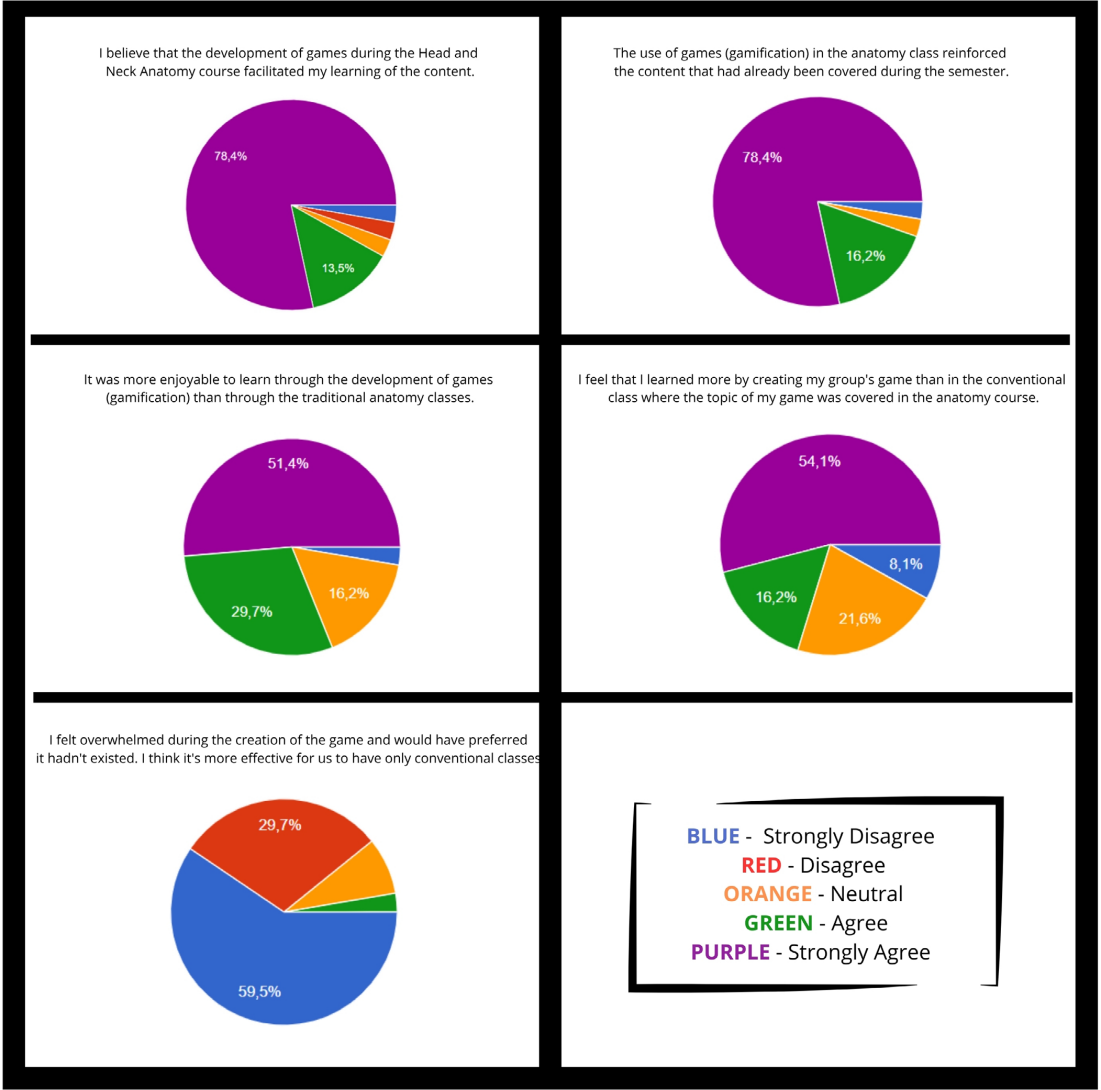
Charts illustrating the percentage of students corresponding to each response category on the Likert scale.

**FIGURE 3:**
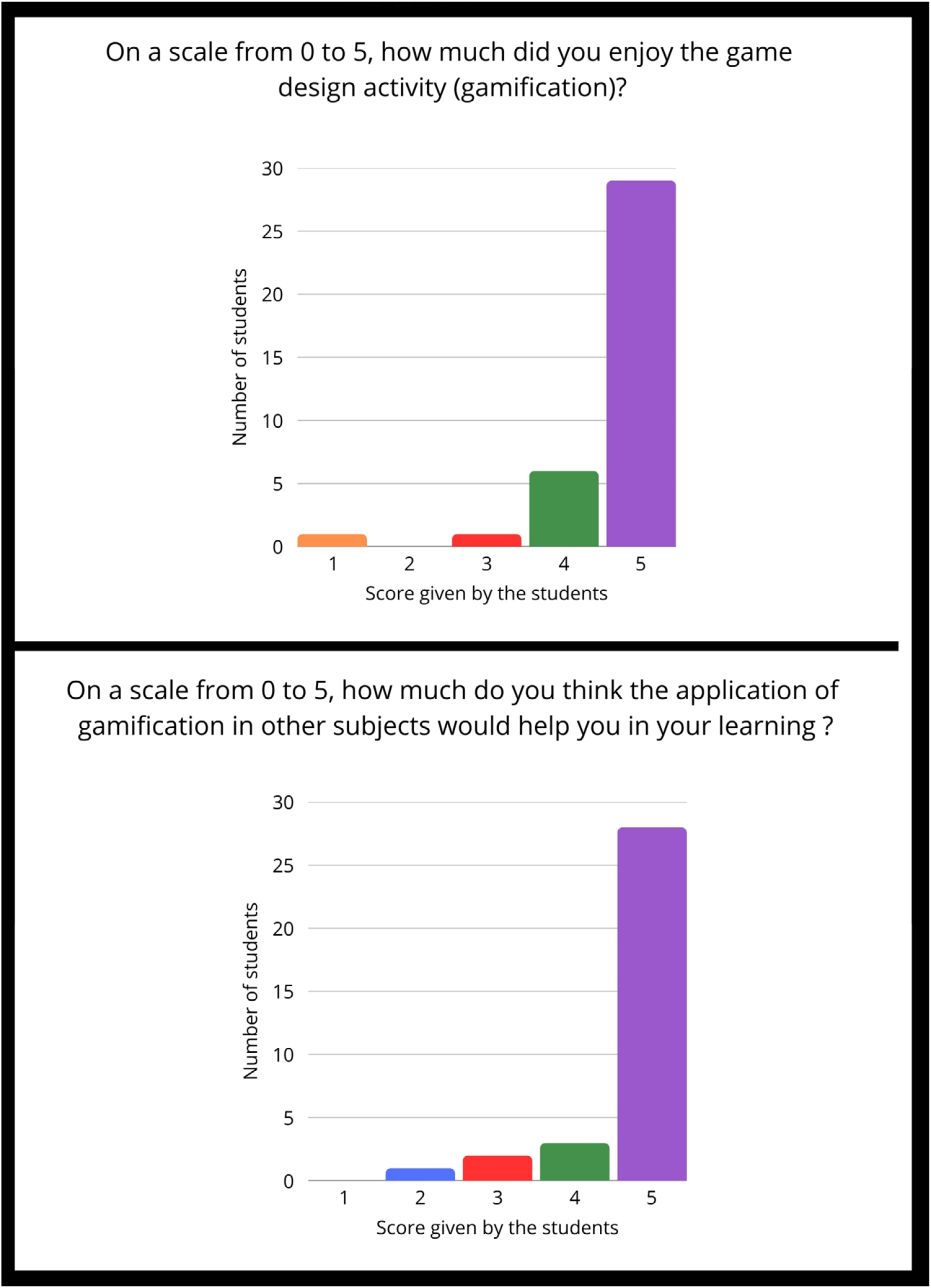
Graphs representing students' opinions on a scale from 1 to 5. In the first graph, 1 indicates that the student *did not enjoy the gamification activity*, while 5 indicates that they *enjoyed it very much*. In the second graph, 1 means the gamification activity *did not help their learning at all*, and 5 means it *helped a lot*.

A total of 70.3% of students felt they learned more during the game development process than in traditional anatomy classes. Nearly 90% of students reported not feeling overwhelmed by the task of creating the games (Figure 2). On a scale from 0 to 5, 78.4% of students rated the activity as a 5, while 16.2% gave it a 4. Again on a scale of 0 to 5, 75.7% of students believed that applying gamification to other subjects would help their learning (Figure 3).

The strengths of this gamification activity included the clear engagement of students in the development of the games, with content assimilation being evident during their presentations. The drawbacks were that some students contributed more than others to the game creation, making it difficult to measure individual levels of participation. To address this, future activities will use smaller groups. The creation of educational games proved to be a practical and accessible strategy that made complex Anatomy content more dynamic, highlighting that educational innovation can stem from creativity and student‐centered approaches rather than expensive technology.

## Conflicts of Interest

The author declares no conflicts of interest.
